# Vitamin D dose response is underestimated by Endocrine Society's Clinical Practice Guideline

**DOI:** 10.1530/EC-13-0008

**Published:** 2013-04-12

**Authors:** Malachi J McKenna, Barbara F Murray

**Affiliations:** 1 St Michael's Hospital, Dún Laoghaire Dublin Ireland; 2 Metabolism Laboratory St Vincent's University Hospital Dublin Ireland; 3 School of Medicine and Medical Sciences University College Dublin DublinIreland

**Keywords:** vitamin D dose response, Institute of Medicine, Endocrine Society's Clinical Practice Guideline

## Abstract

**Objective:**

The recommended daily intakes of vitamin D according to the recent Clinical Practice Guideline (CPG) of the Endocrine Society are three- to fivefold higher than the Institute of Medicine (IOM) report. We speculated that these differences could be explained by different mathematical approaches to the vitamin D dose response.

**Methods:**

Studies were selected if the daily dose was ≤2000 IU/day, the duration exceeded 3 months, and 25-hydroxyvitamin D (25OHD) concentrations were measured at baseline and post-therapy. The rate constant was estimated according to the CPG approach. The achieved 25OHD result was estimated according to the following: i) the regression equation approach of the IOM; ii) the regression approach of the Vitamin D Supplementation in Older Subjects (ViDOS) study; and iii) the CPG approach using a rate constant of 2.5 (CPG2.5) and a rate constant of 5.0 (CPG5.0). The difference between the expected and the observed 25OHD result was expressed as a percentage of observed and analyzed for significance against a value of 0% for the four groups.

**Results:**

Forty-one studies were analyzed. The mean (95% CI) rate constant was 5.3 (4.4–6.2) nmol/l per 100 IU per day, on average twofold higher than the CPG rate constant. The mean (95% CI) for the difference between the expected and observed expressed as a percentage of observed was as follows: i) IOM, −7 (−16,+2)% (*t*=1.64, *P*=0.110); ii) ViDOS, +2 (−8,+12)% (*t*=0.40, *P*=0.69); iii) CPG2.5, −21 (−27,−15)% (*t*=7.2, *P*<0.0001); and iv) CPG5.0+3 (−4,+10)% (*t*=0.91, *P*=0.366).

**Conclusion:**

The CPG ‘rule of thumb’ should be doubled to 5.0 nmol/l (2.0 ng/ml) per 100 IU per day, adopting a more risk-averse position.

## Introduction

Two conflicting reports on vitamin D intake requirements were published in 2011: Institute of Medicine (IOM) report on *Dietary Reference Intakes for Calcium and Vitamin D* and the Endocrine Society's Clinical Practice Guideline (CPG) on Evaluation, Treatment, and Prevention of Vitamin D Deficiency (1, 2). The IOM, on behalf of the USA and Canadian governments, was tasked to review data on calcium and vitamin D intake requirements and their roles in human health (1). The CPG set its objective to provide guidelines to clinicians with a particular emphasis on the care of patients who are at risk for deficiency (2).

IOM specifies an estimated average requirement (EAR) of 400 IU/day for those with minimal or no sunlight exposure – namely, those at risk of privational vitamin D deficiency (3, 4, 5). CPG recommends an intake for those deemed to be at risk that is three- to fivefold higher at 1500–2000 IU/day without any specification about sunlight exposure (2, 6). CPG considers conditions of risk of vitamin D deficiency in need of augmented intakes, but IOM considers that these individuals are at increased risk if sun deprived and are therefore within the realm of the IOM specifications (5). IOM demonstrated that the evidence of benefit plateaus at 30–40 nmol/l (12–16 ng/ml) and covers the majority at 50 nmol/l (20 ng/ml). CPG claims a 25-hydroxyvitamin D (25OHD) threshold of 75 nmol/l (30 ng/ml) as necessary for bone health. Conceptually, IOM deems a 25OHD concentration as a measure of risk of skeletal disease, but CPG deems a 25OHD concentration as diagnostic of ‘deficiency’ or ‘insufficiency’. Operationally, IOM specifies that there is a distribution of requirements called the dietary reference intakes that correspond to 25OHD concentrations: the EAR, which corresponds to 40 nmol/l (16 ng/ml), meets the needs of 50% of the population and the recommended daily allowance, which corresponds to 50 nmol/l (20 ng/ml), meets the needs of all but 97.5% of the population (1, 4, 7). CPG designates 75 nmol/l (30 ng/ml) as the optimal 25OHD concentration for all.

According to the CPG, the vitamin D dose response is best described by a rate constant, or ‘rule of thumb’, whereby 25OHD is expected to increase by 2.5 nmol/l (1 ng/ml) for each 100 IU/day of vitamin D ingested (2, 8). IOM noted a curvilinear response between vitamin D intake and 25OHD as follows: 25OHD nmol/l=9.9×ln(total vitamin D intake (IU/day)). In a study of low-dose oral vitamin D intake (800 IU/day) administered to institutionalized elderly for 16 months with severe hypovitaminosis D, we noted a dose–response of 9.1 nmol/l (3.6 ng/ml) per 100 IU per day, nearly fourfold higher than the CPG estimate (9, 10). Using the IOM regression equation, the predicted mean 25OHD for our study should have been 66 nmol/l (26 ng/ml), which is similar to the observed mean value of 79 nmol/l (31.9 ng/ml) (10). We speculated that the CPG approach by underestimating the vitamin D dose response could be a reason for their higher intake specifications.

## Materials and methods

We only selected studies that had been compiled from the three major reports on vitamin D: Agency for Health Research Quality (AHRQ)–Ottawa, Effectiveness and Safety of Vitamin D in Relation to Bone Health (11); AHRQ–Tufts, Vitamin D and Calcium: Systematic Review of Health Outcomes (12); and the IOM report (1). Studies were chosen in this way because all studies are described in detail, including a critical appraisal and a grading of quality (1, 11, 12). Inclusion criteria for selection of studies were as follows: daily oral dose of vitamin D (D_2_ or D_3_) ≤2000 IU/day; duration at least 3 months; and results of both baseline and post-therapy 25OHD concentrations.

The rate constant for each study was calculated and presented according to the CPG approach of nanomoles per litre rise in 25OHD per 100 IU/day of vitamin D dose. The ratio of observed-to-expected rate constant for each study was calculated. The achieved 25OHD result was estimated according to i) the regression equation approach of the IOM; ii) the regression approach of Vitamin D Supplementation in Older Subjects (ViDOS) (25OHD nmol/l=54.5+24.6×dose/1000−2.5×dose^2^/1000^2^) (13); and iii) the CPG approach using a rate constant of 2.5 (CPG2.5) and a rate constant of 5.0 (CPG5.0). The difference between the expected (*E*) and observed (*O*) was expressed as a percentage of observed and was calculated as follows for each study: ((*E−* *O*)/*O*)×100.

Descriptive statistics are presented as mean and 95% CIs, as median and interquartile range (IQR), or as number and percentage. A one-sample *t*-test was performed to test whether mean differences, as calculated earlier, were different from 0% for each of the four groups. Statistics were performed using IBM SPSS Stats for Windows Version 20.

## Results

Forty-one studies met the selection criteria (Table 1) (14, 15, 16, 17, 18, 19, 20, 21, 22, 23, 24, 25, 26, 27, 28, 29, 30, 31, 32, 33, 34, 35, 36, 37, 38, 39, 40, 41, 42, 43, 44, 45, 46, 47, 48, 49, 50, 51, 52). Studies included young adults (*n*=3), community-dwelling older adults (*n*=22), and institutionalized elderly adults (*n*=16). The majority (*n*=36) were obtained from AHRQ–Ottawa, and 5 were identified from AHRQ–Tufts (16, 17, 18, 20, 52). No additional study was identified in IOM, excluding those studies that were used for the simulated vitamin D dose response. Six studies had two subgroups that were given exactly the same dose; averages of the baseline and post-therapy 25OHD concentrations were calculated rather than have duplicate entries (24, 26, 34, 35, 48, 50). Thirty-three of the studies were randomized control studies regarding the effect of vitamin D supplementation on 25OHD concentrations.

The median (minimum–maximum) dose was 800 (200–2000) IU/day. The median (minimum–maximum) duration of treatment was 12 (3–60) months. The isoform of administered vitamin D was vitamin D_2_ (*n*=1), vitamin D_3_ (*n*=33), and not specified (*n*=7). The median (IQR) 25OHD concentration pre-therapy was 39 (24–61) nmol/l (16 (10–24) ng/ml) and post-therapy was 72 (61–86) nmol/l (29 (24–34) ng/ml).

**Figure 1:**
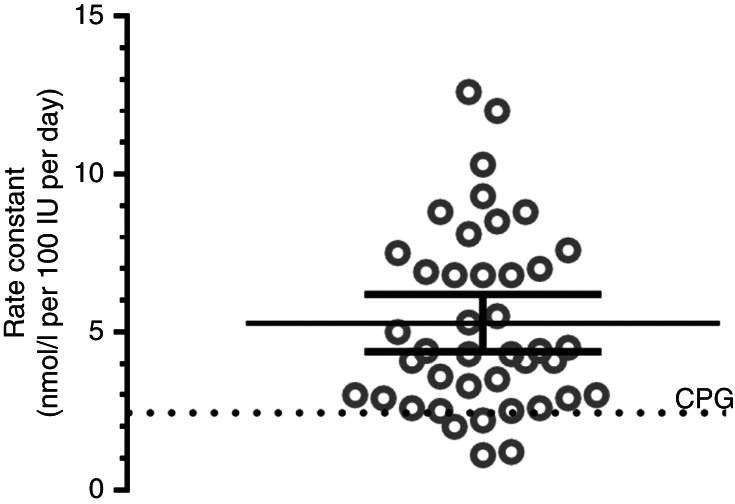
This plot depicts rate constants in the 41 studies. The mean (95% CI) is 5.3 (4.4–6.2) rise of 25OHD nmol/l per vitamin D intake of 100 IU/day. The Clinical Practice Guideline (CPG) rate constant of 2.5 nmol/l per 100 IU per day is depicted by the broken line.

**Figure 2:**
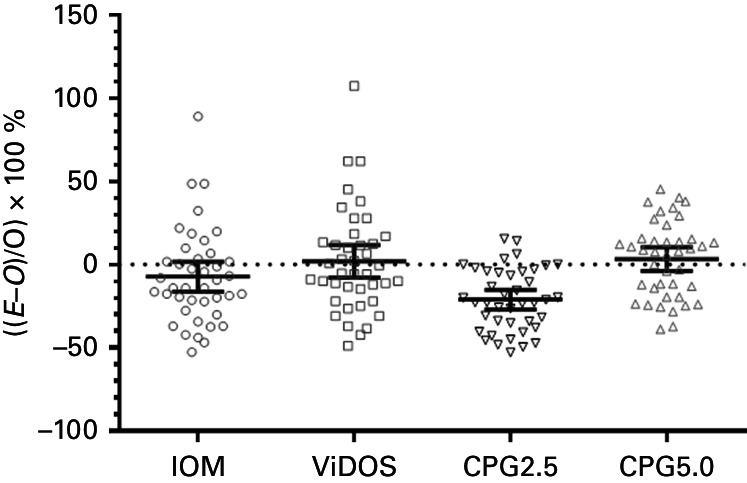
This plot depicts differences between the expected and observed expressed as a percentage of observed in the 41 studies. The mean and 95% CIs are represented by continuous lines. IOM refers to Institute of Medicine report; ViDOS refers to Vitamin D in Older Subjects study; CPG2.5 refers to Clinical Practice Guideline using rate constant of 2.5 nmol/l per 100 IU per day; and CPG5.0 refers to Clinical Practice Guideline using rate constant of 5.0 nmol/l per 100 IU day. E, expected 25OHD; O, observed 25OHD.

The mean (95% CI) rate constant was 5.3 (4.4–6.2) nmol/l per 100 IU per day ranging from 1.1 to 12.6 nmol/l per 100 IU per day (Fig. 1). The mean (95% CI) for the observed:expected ratio of the rate constants with respect to the CPG rate constant of 2.5 nmol/l per 100 IU per day was 2.1 (1.7–2.5). The mean (95% CI) for the difference between the expected and observed expressed as a percentage of observed with the result of the one-sample *t*-tests was as follows: i) for IOM −7 (−16,+2)% (*t*=1.64, *P*=0.110); ii) for ViDOS +2 (−8,+12)% (*t*=0.40, *P*=0.69); iii) for CPG using rate constant of 2.5 was −21 (−27,−15)% (*t*=7.2, *P*<0.0001); and iv) for CPG using rate constant of 5.0 was +3 (−4,+10)% (*t*=0.91, *P*=0.366) (Fig. 2).

## Discussion

The CPG approach is an easy-to-remember ‘rule of thumb’ whereby the clinician calculates the difference between a patient's 25OHD result and the CPG target of 75 nmol/l (30 ng/ml), then divides that difference by their rate constant of 2.5, and finally multiples the answer by 100 to estimate the required vitamin D dose (2, 8). According to the findings of our report, this CPG rate constant on average underestimates the rate constant by twofold. The reason for the substantial underestimate is explained by the dose–response curve for vitamin D. Both IOM and ViDOS noted a curvilinear dose–response curve. The CPG rate constant is principally influenced by a dose–response study in which the baseline 25OHD concentration ∼70 nmol/l (28.0 ng/ml) and three high-dose vitamin D schedules were administered, namely 1000, 5000, and 10 000 IU/day (31). When the IOM was deliberating on its approach to vitamin D dose response, it reviewed previous attempts at estimating a rate constant (11, 31). IOM noted that lower intakes had a greater response, but they also concluded that if an individual was already taking 1000 IU/day, then the rate constant would be ∼2.5 nmol/l (1.0 ng/ml) per 100 IU per day. Another important factor is the degree of hypovitaminosis D: the lower the 25OHD concentration, the greater the response. So the current CPG rate constant should only give an accurate estimate in circumstances when the baseline concentration of 25OHD exceeds 70 nmol/l (28.0 ng/ml) and the intake exceeds 1000 IU/day. Regarding other confounders of the dose–response, the ViDOS study demonstrated that BMI was a confounder with 25OHD response being attenuated by increased BMI; also there was an interaction effect between BMI and time (13). Other covariates had no effect such as age, calcium intake, smoking status, alcohol use, average caffeine intake, and serum creatinine. The IOM report also excluded an interaction effect with age over a broader age range from childhood to the elderly (1).

While we demonstrated a very high rate constant in our study of institutionalized patients at 9.1 nmol/l (3.6 ng/ml) per 100 IU per day, in a subsequent systematic review of published literature up to 1995, we suggested that the average rate constant was 5.5 nmol/l (2.2 ng/ml) per 100 IU per day, which is remarkably similar to the current observation (53). This fact had been noted and discussed by the authors of the study that formed the basis of the CPG rate constant (31). The current finding regarding the rate constant is supported by a meta-regression analysis of randomized control trials of vitamin D supplementation (*n*=51) that has just be published in abstract form (54). The authors noted a mean increase of 48 nmol/l (19.2 ng/ml) with a daily dose of 800 IU/day after 6 months that is equivalent to a rate constant of 6 nmol/l (2.4 ng/ml) per 100 IU per day. Similarly, in a recent systematic review, Autier *et al*. (55) estimated that an intake of 800 IU/day combined with calcium in those with a mean 25OHD level of 25 nmol/l should elevate the level on average by 36 nmol/l, which is equivalent to a rate constant of 4 nmol/l (1.6 ng/ml) per 100 IU per day.

The regression approach, as used by IOM and ViDOS, is much more satisfactory. Both recommend that one should attempt to estimate the target 25OHD concentration based on either total daily oral vitamin D intake according to IOM or on dose administered according to ViDOS. The average observed 25OHD concentration was within the confidence limits according to the 25OHD concentration estimated by both the IOM and ViDOS equations, although the 95% CIs are large. The IOM regression equation slightly underestimates the achieved 25OHD concentration, but this is not unexpected as the IOM regression equation is based on total vitamin D intake and the studies only provided information on vitamin D dose, thus underestimating the total oral vitamin D intake. Regarding a similar analysis of the CPG approach, if a rate constant of 5.0 nmol/l (2.0 ng/ml) per 100 IU per day is chosen instead of a rate constant of 2.5 nmol/l (1.0 ng/ml) per 100 IU per day, then the CPG approach is as good at estimating the 25OHD achieved concentration as both IOM and ViDOS (Fig. 2).

While classical toxicity occurs at 25OHD concentrations above 250 nmol/l (100 ng/ml) (2), there are concerns about harm at much lower concentrations (1, 56). There are emerging concerns about risks at serum 25OHD concentrations above 125 nmol/l (50 ng/ml) (1, 3). There is a substantial safety window between 50 nmol/l (20 ng/ml) and 125 nmol/l (50 ng/ml). There are now five reasons why the Endocrine Society's CPG could lead to either unnecessary overreplacement for many or hypervitaminosis D with potential harm for some: i) labeling patients as ‘deficient’ or ‘insufficient’ rather than viewing a 25OHD concentration as a measure of risk, thus heightening concern; ii) setting a higher threshold for 25OHD at 75 nmol/l (30 ng/ml) compared with 50 nmol/l (20 ng/ml) for IOM; iii) advising that all have 25OHD concentrations above the threshold of 75 nmol/l (30 ng/ml), instead of considering that there is a range of requirements like IOM, which specifies that a concentration above 40 nmol/l (16 ng/ml) meets the needs of 50% of the population according to a probabilistic model (7); iv) failing to distinguish between those ‘at risk’ for privational hypovitaminosis D, whose intake requirements are covered by IOM specifications, and those ‘at risk’ for disease-specific reasons; v) and underestimating the rate constant by twofold that is likely to overestimate the intake requirements in those whose concentrations are below 70 nmol/l (28.0 ng/ml) and whose intakes are below 1000 IU/day.

One example whereby CPG may lead to toxicity is in infancy. CPG recommends intakes of 400–1000 IU/day for all infants, and 2000 IU/day for 6 weeks for those with concentrations below 50 nmol/l (20 ng/ml) (2). IOM, due to lack of evidence, only specifies an ‘adequate intake’ of 400 IU/day, which is likely to meet the needs of the majority (1). In a recent survey of preterm infants with 25OHD concentrations <50 nmol/l (20.0 ng/ml) who were followed into infancy at about 3–4 months, we observed that an intake of 400 IU/day from feeds and supplements yielded an average 25OHD concentration of 83 nmol/l (33 ng/ml). Nearly 10% had concentrations above 125 nmol/l (50 ng/ml), and one infant had a 188 nmol/l (75 ng/ml) who was actually ingesting 850 IU/day, which is within the CPG recommendation (57). There is a recent case series of infants with hypercalcemia highlighting the problem of oversupplementation (58). Infants are most at risk of vitamin D toxicity due to mutations in the vitamin D-metabolizing enzyme CYP24A1 that increases sensitivity to oral vitamin D (59).

IOM has shifted the paradigm from thinking about ‘more is better’ to a more risk-averse approach (3). It has also challenged the notion that harm should just be viewed in terms of vitamin D toxicity such as hypercalcemia, hypercalciuria, or metastatic calcification. It has advanced the concept of ‘harm’ in terms of chronic disease outcomes and mortality (1). This viewpoint is further enhanced by more recent reports on links with all-cause mortality and with prostate cancer (56, 60). Empiric evidence requires demonstration of harm in the setting of a randomized clinical trial. It may be some time before such evidence is forthcoming, but a recent report from Australia is informative. In a randomized trial of annual high-dose oral vitamin D that had falls and fractures as outcome measures, intervention resulted in increased risk of falls and fractures; in a small sample of the treated group, 25OHD levels reached an average concentration of 120 nmol/l that approximates the upper safe level specified by IOM. It is more risk averse to adopt a stochastic approach of harm rather than a deterministic approach of toxicity.

A limitation of this paper is that original studies were not reviewed by us, but instead the data were extracted from three major reports. In deference to the AHRQ and IOM process, it would not have been possible to emulate the work of the Evidence-based Practice Centers that assimilated nearly 40 years of clinical studies on vitamin D, informing their comprehensive assessments. Furthermore, this paper was not designed as a meta-regression analysis. In fact, it started as a clinical observation that the Endocrine Society's approach to vitamin D dose response was far removed from our clinical and research observations and was also inclined substantially toward underestimating the vitamin D response. Another limitation of this study is comparing reports that use different models of the vitamin D dose response: a linear model with two curvilinear models.

It seems prudent to probe the boundaries of benefit by augmenting vitamin D intake to higher levels in carefully conducted research studies, but clinical practice and clinical guidelines need not leap ahead of the evidence as presented in recent reports from AHRQ, IOM, and the US Preventative Services Task Force (1, 11, 12, 61, 62). The way forward is the implementation of IOM recommendations, worldwide, especially given that the new specifications have increased two- to threefold for children and young adults and increased by 33–50% for those over age 50 years compared with the last IOM report in 1997 (63). We conclude that the CPG advice regarding vitamin D dose to patients overestimates the rate constant by twofold on average. We suggest that the ‘rule of thumb’ of the CPG, if it is to be used, should be doubled to 5.0 nmol/l (2.0 ng/ml) per 100 IU per day. This would be more reliable as well as being a more risk-averse approach.

## Author contribution statement

The authors' responsibilities were as follows: M J McKenna conceived the study, analyzed the data, and performed the statistical analyses; M J McKenna and B F Murray wrote the manuscript. Both authors read and approved the final manuscript.

## Figures and Tables

**Table 1:** Studies of low-dose daily vitamin D supplementation drawn from AHRQ–Ottawa report and from AHRQ–Tufts report

**Study**	**Country**	**Group** a	**Dose** (IU/day)	**Duration** (months)	**25OHD** (nmol/l)		**Rate constant** (nmol/l per 100 IU per day)	**Expected 25OHD** (nmol/l)			
Basal	Post	IOM	ViDOS	CPG2.5	CPG5.0
Aloia (14)	USA	2	800	3	48	71	2.0	66	73	68	88
Bischoff-Ferrari (15)	Switzerland	3	800	4	31	66	4.4	66	73	51	71
Bjorkman (16)	Finland	3	400	6	23	50	6.8	59	64	33	43
Bjorkman (16)	Finland	3	1200	6	23	72	4.1	70	80	53	83
Blum (17)	USA	2	700	12	73	122	7.0	65	70	91	108
Bolton-Smith (18)	UK	2	400	60	60	72	3.0	59	64	70	80
Brazier (19)	France	2	800	3	23	65	5.3	66	73	43	63
Bunout (20)	Chile	2	800	12	40	73	4.1	66	73	60	80
Chapuy (21)	France	3	800	18	40	105	8.1	66	73	60	80
Chapuy (22)	France	3	800	24	22	77	6.9	66	73	42	62
Chel (23)	The Netherlands	3	400	4	23	60	9.3	59	64	33	43
Dawson-Hughes (24)	USA	2	700	36	77	112	4.1	65	70	95	112
Deroisy (25)	Belgium	2	200	3	28	43	7.5	52	59	33	38
Deroisy (26)	Belgium	3	800	12	50	111	9.0	66	73	70	90
Goussos (27)	USA	2	800	3	48	64	2.0	66	73	68	88
Grados (28)	France	2	800	12	18	72	6.8	66	73	38	58
Grant (29)	UK	2	800	60	39	62	2.9	66	73	59	79
Harwood (30)	UK	3	800	12	29	50	2.6	66	73	49	69
Heaney (31)	USA	1	1000	5	72	84	1.8	68	77	97	122
Heikkinen (32)	Finland	2	300	12	28	38	3.3	56	62	36	43
Hunter (33)	UK	2	800	24	71	105	4.3	66	73	91	111
Jensen (34)	USA	2	400	36	41	82	9.0	59	64	51	61
Kenny (36)	USA	2	1000	6	65	87	2.2	68	77	90	115
Kenny (35)	USA	2	400	3	61	71	1.5	59	64	71	81
Komulainen (37)	Finland	2	300	6	29	38	3.0	56	62	37	44
Kreig (38)	Switzerland	3	880	24	30	66	4.1	67	74	52	74
Lips (39)	The Netherlands	2	400	36	27	54	6.8	59	64	37	47
Lips (40)	The Netherlands	3	400	12	24	72	12.0	59	64	34	44
Lips (40)	The Netherlands	3	800	12	24	85	7.6	66	73	44	64
Lovell (41)	Australia	3	230	3	18	47	12.6	54	60	24	29.5
Lovell (41)	Australia	3	866	3	41	78	4.3	67	74	63	84.3
Meier (42)	Australia	2	500	24	75	88	2.6	62	66	88	100
Ooms (43)	The Netherlands	2	400	24	27	62	8.8	59	64	37	47
Orwoll (44)	USA	2	1000	12	60	85	2.5	68	77	85	110
Patel (45)	UK	1	800	12	68	77	1.1	66	73	88	108
Riis (46)	Denmark	2	2000	12	33	120	4.4	75	94	83	133
Schaafsma (47, 48)	The Netherlands	2	400	12	90	125	11.0	59	64	100	110
Sebert (49)	Finland	3	800	6	7	35	3.5	66	73	27	47
Sorva (50)	Finland	3	1000	9	12	57	4.4	68	77	37	62
Vieth (51)	Canada	1	600	6	46	79	5.5	63	68	61	76
Zhu (52)	Australia	2	1000	60	68	104	3.6	68	77	93	118

a Groups: 1, young adults; 2, community-dwelling older adults and elderly; 3, institutionalized elderly adults. IOM refers to Institute of Medicine report; ViDOS refers to Vitamin D in Older Subjects study; CPG2.5 refers to Clinical Practice Guideline using rate constant of 2.5 nmol/l per 100 IU per day and CPG5.0 refers to Clinical Practice Guideline using rate constant of 2.5 nmol/l per 100 IU per day. Conversion factor for 25OHD: 1 ng/ml=2.5 nmol/l.
